# Development of Resistance to Damping-Off in Rice, *Oryza sativa* L., Using CRISPR/Cas9

**DOI:** 10.3390/ijms26199761

**Published:** 2025-10-07

**Authors:** Seung-Kyo Jeong, Jae-Ryoung Park, Eun-Gyeong Kim, Kyung-Min Kim

**Affiliations:** 1 Field Crop Research Division, National Institute of Crop and Food Science, RDA, Miryang 50424, Republic of Korea; tmdry1221@gmail.com; 2 Crop Breeding Division, National Institute of Crop Science, Rural Development Administration, Wanju 55365, Republic of Korea; icd0192@korea.kr; 3 National Agrobiodiversity Center, National Institute of Agricultural Sciences, Rural Development Administration, Jeonju 54874, Republic of Korea; keg950@korea.kr; 4 Coastal Agriculture Research Institute, Kyungpook National University, Daegu 41566, Republic of Korea; 5 Department of Applied Biosciences, Graduate School, Kyungpook National University, Daegu 41566, Republic of Korea

**Keywords:** CRISPR/Cas9, damping-off, genome editing, SDN-1

## Abstract

Damping-off disease hinders rice seedling growth and reduces yield. Current control methods, such as seed or soil sterilization, rely on chemicals that cause environmental pollution and promote pathogen resistance. As a sustainable alternative, we targeted the damping-off resistance-related gene *OsDGTq1* using CRISPR/Cas9. Field experiments first verified *OsDGTq1*’s significance in resistance. The CRISPR/Cas9 system, delivered via Agrobacterium-mediated transformation, was used to edit *OsDGTq1* in rice cultivar Ilmi. Lesions from major damping-off pathogens, *Rhizoctonia solani* and *Pythium graminicola*, were observed on G_0_ plants. All 37 regenerated plants contained T-DNA insertions. Among them, edits generated by sgRNA1-1, sgRNA1-2, and sgRNA1-3 resulted in the insertion of two thymine bases as target mutations. Edited lines were assigned names and evaluated for agronomic traits, seed-setting rates, and pathogen responses. Several lines with edited target genes showed distinct disease responses and altered gene expression compared to Ilmi, likely due to CRISPR/Cas9-induced sequence changes. Further studies in subsequent generations are needed to confirm the stability of these edits and their association with resistance. These results confirm that genome editing of *OsDGTq1* alters resistance to damping-off. The approach demonstrates that gene-editing technology can accelerate rice breeding, offering an environmentally friendly strategy to develop resistant varieties. Such varieties can reduce chemical inputs, prevent pollution, and minimize seedling loss, ultimately enhancing food self-sufficiency and stabilizing rice supply.

## 1. Introduction

Rice (*Oryza sativa* L.) is a crucial staple food for over half the global population [1]. However, significant reductions in rice yields have been seen recently, driven by rapid climate change and increasing pest infestations [2]. Rice is exposed to diverse biotic and abiotic stresses under varying environmental conditions. Stress and pathogen infections during growth and developmental stages threaten rice quality and yield, leading to their reduction [3]. Among them, damping-off is a soil-borne disease that causes significant damage to almost all crops worldwide, particularly important crops such as tomatoes, cucumbers, peppers, squash, and rice. *Rhizoctonia solani*, *Pythium* spp., *Fusarium* spp., *Phytophthora* spp., *and Sclerotinia* spp. have been reported to cause damping-off disease, with *Rhizoctonia solani*, *Pythium graminicola*, and *Pythium ultimum* being the most frequently occurring pathogens [4]. Damping-off disease predominantly occurs in rice seedlings, with conditions such as waterlogged soil, rising soil temperatures after germination, and plant overcrowding ideal for its development; seeds often rot before they can germinate [5,6]. Additionally, if the pathogen infects the roots or stems, growth is inhibited, and the plant may collapse due to a lack of structural support. The lesions become reddish-brown or black and spread rapidly on the young stems. The damping-off pathogens can survive in the soil for long periods, even without a host plant, making thorough preparation essential for its prevention [7]. Therefore, seeds and soil are sterilized or disinfected to prevent or control damping-off disease [8]. However, improper use of chemical agents can lead to environmental pollution and the emergence of resistant pathogens [9]. Thus, breeding resistant crops and researching resistance-related genes are actively being pursued to reduce the use of chemical substances [10]. Methods for suppressing gene expression include RNA interference (RNAi) and CRISPR/Cas9 gene-editing technology. These two technologies share the common feature of using small RNAs with specific target sequences 18–20 nucleotides long [11]. Genome editing through the CRISPR/Cas9 system is mediated by a complex comprising CRISPR RNA (crRNA), trans-activating CRISPR RNA (tracrRNA), and the Cas9 nuclease. This CRISPR/Cas9 complex identifies and binds to complementary DNA sequences flanked by a protospacer-adjacent motif (PAM), facilitating targeted DNA cleavage [12]. CRISPR completely and permanently removes genes at the DNA level, fully blocking protein expression and preventing any disruptions caused by low-level protein expression [13]. In this context, we hypothesize that modifications in the conserved domain of *OsDGTq1*, a gene identified by QTL mapping as being associated with damping-off resistance [14], could affect the defense signaling pathway and alter the resistance of rice seedlings. Therefore, this study aimed to edit the *OsDGTq1* gene using the CRISPR/Cas9 system and evaluate its potential role in conferring resistance to damping-off pathogens.

In this study, *OsDGTq1*, a gene associated with resistance to damping-off, was investigated in Ilmi (*Oryza sativa* L. spp. *japonica* cv. Ilmi). Using the CRISPR/Cas9 system, *OsDGTq1* was homozygously edited in the 0 generation, and transgene-free lines with homozygous edits were subsequently selected. These genome-edited lines were evaluated to determine *OsDGTq1* contributes to resistance against damping-off. The results demonstrate that the application of CRISPR/Cas9 technology enables precise and time-efficient editing of target genes, thereby enhancing disease resistance while providing insights into gene function, highlighting its potential as an effective tool for rice breeding.

## 2. Results

### 2.1. Significance Analysis of OsDGTq1 Through Field Experiments

#### Field Investigation of *OsDGTq1*

**Figure 1:**
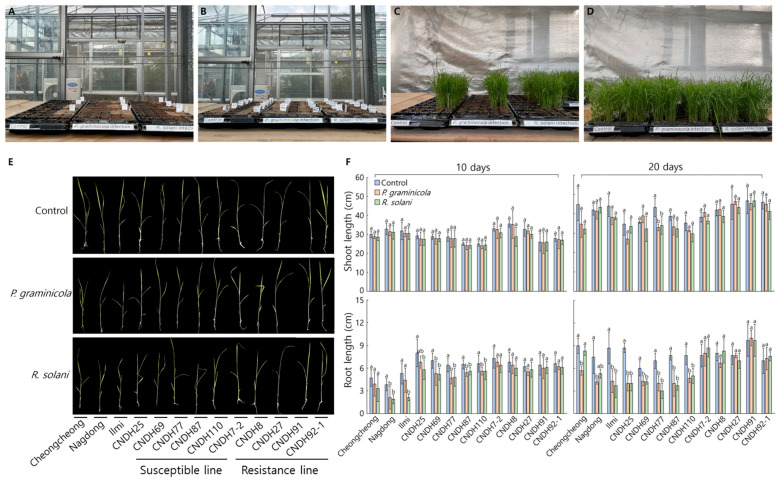
Validation of the Significance of *OsDGTq1* in Field Conditions. (**A**,**B**) Sowing of damping-off susceptible and resistant lines. (**C**,**D**) Plants 11 days after sowing. (**E**) Images of plants 10 days after pathogen inoculation. (**F**) Comparative analysis of plants at 10 days and 20 days post-inoculation. Mean denoted by the same letter are not significantly different (*p* < 0.05) as evaluated by Duncan’s multiple range test (DMRT).

To evaluate the significance of *OsDGTq1* in the field, pathogens were inoculated at the seedling stage into Cheongcheong, Nagdong, Ilmi, the CNDH damping-off resistant line, and the CNDH damping-off susceptible line. Ten days after inoculation (Figure 1A–D), the mean shoot and root lengths of untreated control plants and inoculated experimental plants were compared and analyzed (Figure 1E). For root length measurements, significant differences were observed between the control and pathogen-inoculated groups in the CNDH susceptible line at both 10 and 20 days after inoculation. In contrast, no significant differences were observed in the resistant line under the same conditions (Figure 1F).

### 2.2. CRISPR/Cas9-Mediated Editing of OsDGTq1

#### 2.2.1. SgRNA Design and Development of *OsDGTq1* Genome-Edited Rice Using CRISPR/Cas9

**Figure 2:**
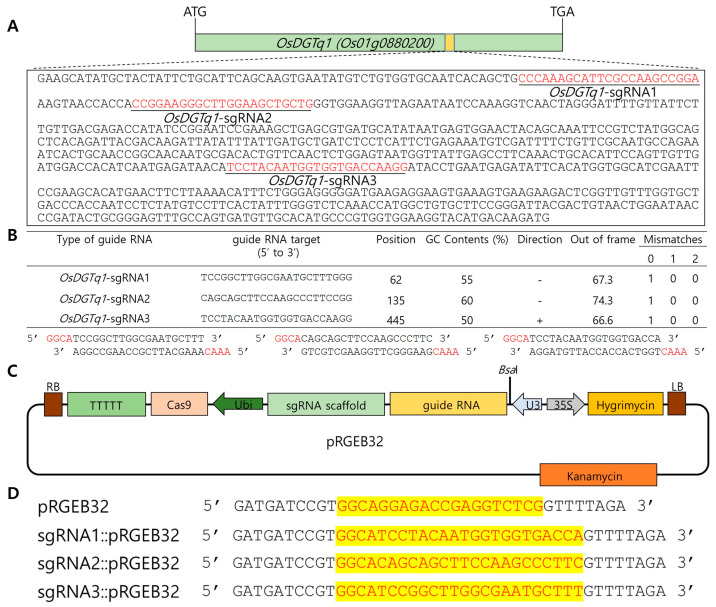
Schematic Diagram of *OsDGTq1* sgRNA, Cas9 Vector, and Construction of the Ligation Vector. (**A**) *OsDGTq1* conserved domain sequence red letters were sgRNA. (**B**) Three sgRNAs were designed for genome editing of *OsDGTq1*. The GC content of the gRNAs was set to 50–60%. The out-of-frame value was set to at least 65, and a mismatch of 1-0-0 meant that the same sequence was not present in the designed gRNA and *OsDGTq1*. (**C**) pRGEB32 vector diagram and pRGEB32 vector used for genome editing of *OsDGTq1* through CRISPR/Cas9 expression in rice. (**D**) Confirmation of sgRNA::pRGEB32 vector plasmid ligation by sequencing.

**Figure 3:**
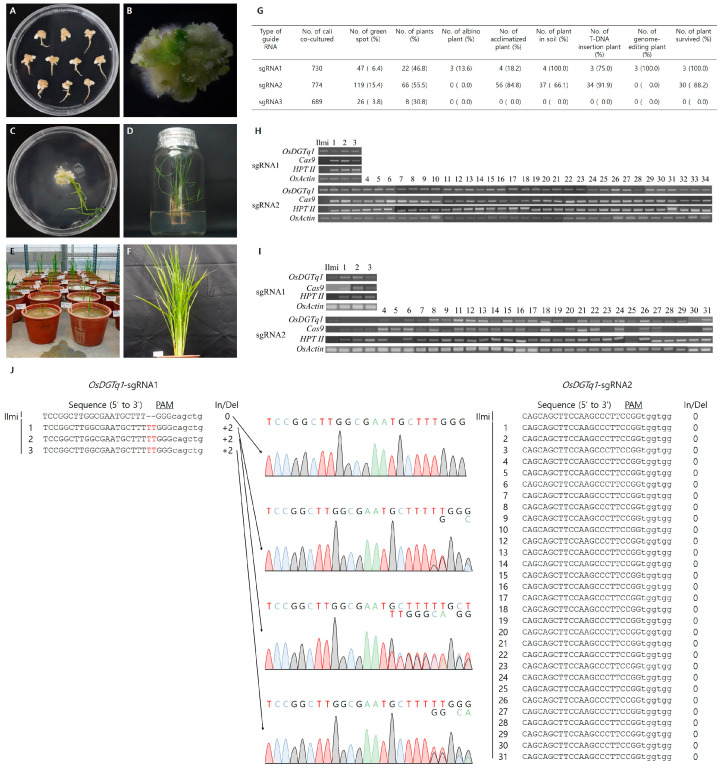
Tissue culture system and development *OsDGTq1* genome-editing rice. (**A**) Calli derived from callus induction media for 21 days was selected and used for *Agrobacterium*-mediated transformation. (**B**) *Agrobacterium* was used to inoculate the callus and green spots formed in the regeneration media after 14 days. (**C**) Shooting and rooting were performed at the green spots. (**D**) Shooting callus was transferred to fresh regeneration media for rooting for 30 days, followed by a 3-day acclimatization treatment with perforated aluminum foil. (**E**) After acclimatization, regeneration plants were transplanted into soil. (**F**) The panicles emerged 60 days after transplantation was performed. (**G**) Efficiency of regeneration plant breeding using CRISPR/Cas9. (**H**) PCR amplification of *OsDGTq1* transferred genes (*Cas9* and *HPT II*) and *OsActin* in regenerated plants. (**I**) RT-PCR amplification of *OsDGTq1,* transferred gene (*Cas9* and *HPT II*) and *OsActin* in regenerated plants. (**J**) Genotyping analysis to identify genome-edited (GE) rice based on sequence alignment with Ilmi at the *OsDGTq1* region, highlighting specific insertions.

The designing of sgRNAs for targeted gene editing was performed using the CRISPR RGEN Tools (http://www.rgenome.net/ (accessed on 23 August 2025)) program. Three sgRNAs were specifically designed to target the domain region of the target gene (Figure 2A,B). In the pRGEB32 vector, the sgRNA is expressed under the control of the U3 promoter; thus, the sgRNA was designed to be inserted at the BsaI restriction enzyme site in the vector (Figure 2C). The pRGEB32 vector was transformed into JM109 competent cells, and the transformed JM109 cells were selected on a LB medium containing kanamycin, the selection marker for pRGEB32. Plasmids were extracted from the selected E. coli, and the insertion of the sgRNA was confirmed through sequencing (Figure 2D). The sgRNA::pRGEB32 plasmid vector was introduced into Ilmi callus via Agrobacterium-mediated transformation. Ilmi callus was used to develop genome-edited lines targeting *OsDGTq1*. Ilmi seeds with uncontaminated and intact embryos were selected for use. A total of 41 plants were regenerated, among which 37 transgenic lines and 3 gene-edited lines were obtained (Figure 3A–G).

#### 2.2.2. Genotype Analysis and Gene Expression Analysis of *OsDGTq1* Regenerated Plants

To confirm the bands of the target gene *OsDGTq1*, the *Cas9*, the selection marker *HPT II*, and *OsActin* in the regenerated plants, DNA was extracted from 37 regenerated plants and Ilmi. The band relating to the target gene *OsDGTq1* was confirmed in all plants. The *Cas9* and *HPT II* bands were confirmed in all regenerated plants, excluding Ilmi. The band for *OsActin* was confirmed in all plants (Figure 3H). After DNA analysis, plants 11, 32, 33, and 34 from the sgRNA2 regenerated plants did not survive. Gene expression was analyzed using 33 regenerated plants and Ilmi, excluding the four that did not survive. After RNA extraction, cDNA synthesis, and PCR were performed to analyze *OsDGTq1*, *Cas9*, *HPT II*, and *OsActin* expressions. This study found that *OsDGTq1* was expressed in all plants with sgRNA1, while for sgRNA2, *OsDGTq1* was in all plants except plants 1, 2, 3, and 5. For sgRNA1, both *Cas9* and *HPT II* were confirmed to be expressed in all plants. Meanwhile, for sgRNA2, *Cas9* was not expressed in plants 25, 28, 29, and 30, and *HPT II* was not expressed in plant 25. Neither *Cas9* nor *HPT II* were expressed in Ilmi. *OsActin* was confirmed to be expressed in all plants of both sgRNA1 and sgRNA2 (Figure 3I). DNA was extracted from the bands of the target gene and analyzed. In the three plants containing sgRNA1, it was confirmed that two thymine (T) bases were inserted into the target sequence. For the 34 plants with sgRNA2, it was confirmed that the target sequence was identical to that of Ilmi (Figure 3J).

#### 2.2.3. Nomenclature of G_0_ Transgenic Lines and Evaluation of Agronomic Traits

The transgenic lines are from the G_0_ generation. Each G_0_ plant was established as an independent line. Starting with the first plant of sgRNA1, they were named *OsDGTq1*-G1, and continuing up to *OsDGTq1*-G34, excluding the missing 11th plant of sgRNA2, *OsDGTq1*-G14. The agricultural traits (plant height (cm), culm length (cm), panicle length (cm), number of tillers, and number of spikes) of regeneration plants that occurred genome-editing of *OsDGTq1* in 0 generations (G_0_) were investigated (Table S1). The fertilization rate was calculated by dividing the number of filled grains by the total number of spikelets and multiplying by 100 (Table S2).

#### 2.2.4. Analysis of Target Gene Expression Levels in Genome-Edited Lines

**Figure 4:**
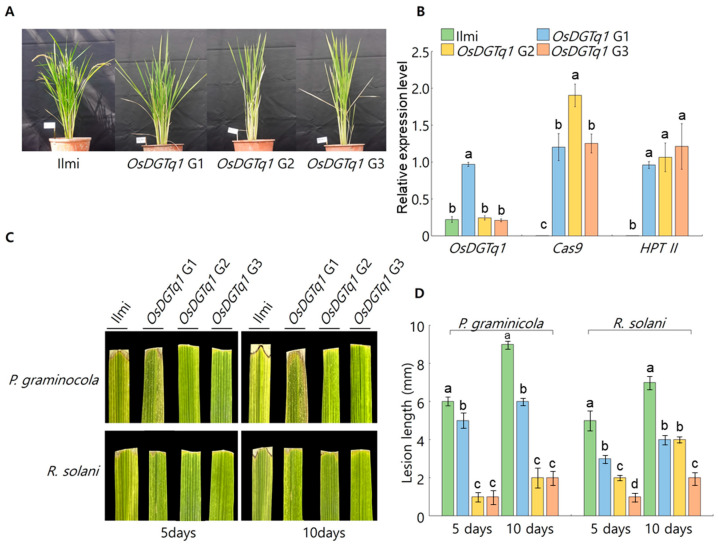
Analysis of target gene expression levels and damping-off disease resistance in genome-editing rice and Ilmi using leaves from Genome-editing plants. (**A**) Wild-type Ilmi and genome-edited rice. (**B**) Expression levels of target genes *OsDGTq1*, *Cas9*, and *HPT II*. (**C**,**D**) Lesion length analysis on leaves of Ilmi and genome-edited rice inoculated with damping-off pathogens 5 days and 10 days post-inoculation. Mean denoted by the same letter are not significantly different (*p* < 0.05) as evaluated by Duncan’s multiple range test (DMRT).

qRT-PCR was performed to evaluate the expression levels of *OsDGTq1*, *Cas9* and *HPT II* in genome-edited lines (Figure 4A,B). Ilmi, *OsDGTq1* G2, and *OsDGTq1* G3 exhibited similar expression levels, while *OsDGTq1* G1 showed a distinct difference. *Cas9* and *HPT II* were not expressed in Ilmi. *Cas9* showed similar expression levels between *OsDGTq1* G1 and *OsDGTq1* G3, while *HPT II* exhibited comparable expression levels across *OsDGTq1* G1, *OsDGTq1* G2, and *OsDGTq1* G3. 

#### 2.2.5. Predicted Structural Consequences of Frameshift Mutation in OsDGTq1

Sequence analysis revealed that the wild-type allele encodes a complete protein of 635 amino acids. In contrast, the mutant allele contained two thymine (T) insertions within the coding region, which caused a frameshift starting from approximately the 328th codon. This frameshift introduced a premature stop codon (TAA), resulting in a truncated protein of 346 amino acids. Consequently, the mutant protein lacks 289 amino acids at the C-terminal region compared with the wild type.

#### 2.2.6. Investigation of G_0_ Genome-Edited Lines Leaf Response to Damping-Off Pathogen Strains

After dipping the scissors in the culture medium, the third leaf was cut to inoculate the pathogen, and the lesion lengths were assessed twice, five days and ten days after inoculation. The lesion lengths were measured at the longest point (Figure 4C,D). In Figure 4C, the most representative phenotypes of lesion length caused by *P. graminicola* and *R. solani* in each line are presented. Five days after inoculation with P. graminicola, the lesion lengths were as follows. Ilmi was 6 ± 0.13mm, *OsDGTq1* G1 was 5 ± 0.41 mm, *OsDGTq1* G2 was 1 ± 0.31 mm, and *OsDGTq1* G3 was 1 ± 0.25 mm. Five days after inoculation with *R. solani*, the lesion lengths were measured: Ilmi was 5 ± 0.21 mm, *OsDGTq1* G1 was 3 ± 0.22 mm, *OsDGTq1* G2 was 2 ± 0.15 mm, and *OsDGTq1* G3 was 1 ± 0.18 mm. Ten days after inoculation with *P. graminicola*, the lesion lengths were as follows. Ilmi was 9 ± 0.21 mm, *OsDGTq1* G1 was 6 ± 0.19 mm, *OsDGTq1* G2 was 2 ± 0.14 mm, and *OsDGTq1* G3 was 2 ± 0.15 mm. Ten days after inoculation with *R. solani*, the lesion lengths were measured: Ilmi was 7 ± 0.16 mm, *OsDGTq1* G1 was 4 ± 0.13 mm, *OsDGTq1* G2 was 4 ± 0.12 mm, and *OsDGTq1* G3 was 2 ± 0.19 mm.

## 3. Discussion

Rice is one of the world’s three major staple crops and serves as a primary food source, particularly in Asia [15]. However, rice yields are declining due to the reduction in agricultural land area and the impact of pests and diseases exacerbated by abnormal temperature fluctuations [16].

In this study, the *OsDGTq1* gene, identified by QTL mapping, was edited using the CRISPR/Cas9 system to evaluate resistance against the major damping-off pathogens *P. graminicola* and *R. solani* [4,17]. Stable rice production is increasingly uncertain due to population growth [18], declining grain quality [19], and multiple biotic and abiotic stresses [20]. Understanding genetic resistance and developing resistant cultivars remain essential goals [21,22,23]. Our findings provide fundamental insights for future rice breeding strategies targeting disease resistance.

The rationale for employing the CRISPR/Cas9 system in this study was to overcome the limitations of RNA interference (RNAi), which is often associated with transient gene silencing and low stability. By inducing direct mutations at the genomic level, CRISPR/Cas9 offers the advantage of generating resistant lines that can be stably transmitted to subsequent generations. Indeed, successful applications of CRISPR/Cas9 for improving disease resistance and agronomic traits have been reported in several crops, including maize, wheat, and tomato [11], suggesting that this strategy is also promising for rice.

Therefore, it is essential to understand genetic resistance, study genes conferring resistance to damping-off, and develop resistant varieties. In this study, *OsDGTq1*, which was identified through QTL mapping as being involved in resistance to the major damping-off pathogens *P. graminicola* and *R. solani*, was targeted for genome-editing using the CRISPR/Cas9 system. This strategy can contribute to reducing yield loss caused by damping-off disease during the seedling stage. This system is based on SDN-1 technology, which induces DSBs at specific gene locations to edit the gene. [24]. Pathogens were inoculated into the seedlings of Cheongcheong, Nagdong, Ilmi, the CNDH damping-off resistant line, and the CNDH damping-off susceptible line. When comparing shoot lengths after inoculation with *P. graminicola* and *R. solani*, no significant differences were observed across all lines. In the comparison of root lengths after inoculation, the parental line Cheongcheong did not show any significant differences, whereas Nagdong and Ilmi exhibited significant differences. Among the CNDH susceptible lines, CNDH24, CNDH69, CNDH77, and CNDH87 showed significant differences, while CNDH110 did not. In the CNDH resistance lines, no significant differences were observed in any of the lines. The lack of significant differences in shoot length across all lines may be attributed to the fact that *P. graminicola* and *R. solani* are soil-borne pathogens, and the inoculation was performed 10 days after germination, which may have only indirectly affected the shoots [25]. The significant differences observed in root length among the susceptible lines further confirmed the accuracy of the population used to detect *OsDGTq1* [26]. Through this experiment, it was confirmed that both this line and *OsDGTq1* demonstrate significant results not only in controlled environments but also in field conditions.

The *OsDGTq1*-G_0_ plants were developed by editing *OsDGTq1*. When the T-DNA insertion in *OsDGTq1*-G_0_ plants was confirmed, the insertion of *Cas9* and *HPT II* was also observed in all 37 G_0_ plants. In the lines where the insertion of *Cas9* and *HPT II* was confirmed, sequence alignment of the sgRNA target site was analyzed, and indels mutations were identified in three lines (sgRNA1-1, sgRNA1-2, and sgRNA1-3: insertion of two thymine (T) bases). After DNA analysis, the transgenic plants sgRNA2-11, sgRNA2-32, sgRNA2-33, and sgRNA2-34 did not survive. Thus, excluding the four non-viable plants, gene expression was analyzed in the remaining 33 plants. In the sgRNA1 plants, the target gene *OsDGTq1* was expressed in all plants. In the sgRNA2 plants, *OsDGTq1* was not expressed in plants 1, 2, 3, and 5. In the genome-edited sgRNA1 plants, gene expression was observed. However, when using the CRISPR/Cas9 system, mosaicism can occur, which may lead to partial gene expression [27,28]. When using CRISPR/Cas9 system, DSBs are induced, and the sequence is corrected through small insertions or deletions [24]. When DNA damage is induced, the chromatin structure relaxes, allowing repair enzymes to access the site and facilitate the repair process. Even without changes in the sequence, gene expression can be altered through changes in chromatin state [29]. In plants sgRNA 2-1, sgRNA 2-2, sgRNA 2-3, and sgRNA 2-5, which were not edited, the target gene may not have been expressed. Expression analysis revealed that sgRNA1-1, sgRNA1-2, and sgRNA1-3 expressed both *Cas9* and *HPT II*. In contrast, sgRNA2-25 did not express either *Cas9* or *HPT II*. Additionally, sgRNA2-28, sgRNA2-29, and sgRNA2-30 exhibited expression of *HPT II*, while *Cas9* expression was absent. Both *Cas9* and *HPT II* are located in the T-DNA region of pRGEB32 but are driven by different promoters [30]. The integration of T-DNA into the plant genome occurs randomly [31]. When T-DNA is integrated into transcriptionally repressive chromatin, such as heterochromatin [32], gene expression may be suppressed. Furthermore, when inserted into repressive chromatin, *Cas9* and *HPT II* may exhibit differential expression due to being regulated by different promoters [33]. Through this experiment, 37 T-DNA inserted plants were developed, Among the 37 plants, 3 were developed with two Thymine base insertions (sgRNA1-1, sgRNA1-2 and sgRNA1-3). When inoculated with *P. graminicola*, sgRNA1-1, sgRNA1-2, and sgRNA1-3 all showed shorter lesion lengths compared to Ilmi. Similarly, when inoculated with *R. solani*, sgRNA1-1, sgRNA1-2, and sgRNA1-3 all had shorter lesion lengths than Ilmi. When comparing the growth characteristics of the G_0_ generation with those of the background variety, Ilmi, used for gene editing, differences were observed. When plants are developed using the CRISPR/Cas9 system, genetic variations may exist in the early generations due to gene editing. However, as generations progress, agronomic traits other than the target trait tend to become similar to those of the parental line [26]. It is observed that transgenic plants generally exhibit reduced seed set rates. Plants regenerated through tissue culture can express a wide range of altered phenotypes. For example, these may include chlorophyll deficiency, dwarfism, seed characteristics, reproductive structures, and necrotic leaves [34]. Additionally, reductions in spikelet fertility and changes in agronomic traits have been reported in regenerated plants [35,36]. Deletions and insertions of bases, along with changes in chromosome structure and number, can induce phenotypic and genotypic variations in the initial generations of transgenic and gene-edited plants due to the insertion of foreign genes [37]. The expression levels of the target gene, *OsDGTq1*, were analyzed in G_0_ genome-edited lines and the parental line, Ilmi. *OsDGTq1* G1 showed a significant difference in expression compared to Ilmi, whereas *OsDGTq1* G2 and *OsDGTq1* G3 exhibited no significant differences from Ilmi. Inoculation of the major damping-off pathogens, *P. graminicola* and *R. solani*, was performed on the leaves of G_0_ gene-edited plants. Lesion lengths were compared with Ilmi. Five days after *P. graminicola* inoculation, Ilmi exhibited the longest lesion length, while *OsDGTq1* G2 and *OsDGTq1* G3 showed the shortest lesions. Ten days after *P. graminicola* inoculation, Ilmi still had the longest lesion length, with *OsDGTq1* G2 and *OsDGTq1* G3 displaying the shortest lesion lengths. Five days after *R. solani* inoculation, Ilmi showed the longest lesion length, while *OsDGTq1* G3 exhibited the shortest. Ten days after *R. solani* inoculation, Ilmi again showed the longest lesion length, and *OsDGTq1* G3 had the shortest lesion length.

The frameshift mutation caused by the insertion of two thymine nucleotides is predicted to generate a truncated protein lacking approximately 289 amino acids at the C-terminus. Such truncation is generally associated with reduced structural stability, loss of functional domains, or targeting of the aberrant protein to cellular degradation pathways. Given that the C-terminal region often contributes to protein–protein interactions and functional regulation, this mutation is expected to exert a negative impact on normal protein function.

Even though genome editing of *OsDGTq1* resulted in improved resistance to damping-off, a complete understanding of the resistance-related mechanisms remains limited. Despite these limitations, our findings highlight the potential of CRISPR/Cas9 technology as a fast, reliable, and heritable genome-editing approach for rice improvement. By precisely targeting resistance-related genes such as *OsDGTq1*, this strategy can contribute to the development of cultivars resistant to damping-off disease. Such innovations are expected to reduce reliance on chemical control measures, mitigate environmental pollution, and enhance food security in the context of global climate change.

## 4. Materials and Methods

### 4.1. In Planta Functional Analysis of Genes

#### 4.1.1. Isolation of *P. graminocola* and *R. solani*

The major pathogens of damping-off, *P. graminicola* KACC 40155 and *R. solani* KACC 40153, were provided by the Korean Agricultural Culture Collection for accurate research. Each strain was cultured in potato dextrose broth (Bexton, Dickinson and Co., Franklin Lakes, NJ, USA) containing agar under dark conditions at 25 °C for 3 days.

#### 4.1.2. Inoculation with Damping-Off Pathogens in the CNDH Population at the Seedling Stage

To compare and analyze the shoot and root lengths, the damping-off pathogens were inoculated at the seedling stage of the Cheongcheong, Nagdong, Ilmi, and CNDH populations, Ilmi represented the background variety for the development of the genome-edited rice, and the parental lines of the CNDH population. The CNDH group represented the CNDH resistance and susceptible lines. Resistance lines were CNDH-7-2, CNDH-8, CNDH-27, CNDH-91, and CNDH-92-1. Susceptible lines were CNDH-25, CNDH-69, CNDH-77, CNDH-87, and CNDH-110. On 8 September 2024, for pre-germination and seed sterilization, seeds were soaked in a solution formed by diluting 10 mL of SPOTAK (Hankooksamgong, Seoul, Republic of Korea) in 20 L of water. The seeds were fully submerged in the solution for 24 h to ensure sterilization, after which the seeds were removed and replaced in clean water.

### 4.2. CRISPR/Cas9-Mediated Genome Editing

#### 4.2.1. Plant Material

The seeds and plants used in this experiment were cultivated and harvested at the Kyungpook National University GMO field located in Hyoryeong-myeon, Gunwi-gun, Daegu Metropolitan City, South Korea (latitude 36° 6′ 46.13″ N, longitude 128° 38′ 25.21″ E). The Ilmi seeds, used as the background for the gene-edited lines, were cultivated and harvested in 2023. The Cheongcheong, Nagdong and Ilmi varieties, used for inoculation with the damping-off pathogen, were transplanted on 23 May 2024. The seeds of Cheongcheong, Nagdong, Ilmi, CNDHs, and Genome-editing lines used to confirm seedling-stage resistance to damping-off, were cultivated and harvested in 2023.Each line was transplanted in two rows in the field, with 25 plants per row, and a planting distance of 30 × 15 cm was maintained. All plants were cultivated following the Rural Development Administration (RDA) guidelines for agricultural science and technology research, maintaining the standard fertilization rate of N−P_2_O_5_−K_2_O = 9:4.5:5.7 kg/10 a.

#### 4.2.2. Design of Single Guide RNA for Genome Editing

We utilized the third-generation gene-editing technology, the CRISPR/Cas9 system, for genome editing. The single guide RNA (sgRNA), designed to edit the *OsDGTq1* gene, targeted the conserved domain region in the *OsDGTq1* coding sequence (CDS). The sgRNA was created using the CRISPR RNA-guided engineered nuclease (RGEN) tool. We selected the 5′-NGG-3′ site for the protospacer adjacent motif (PAM) sequence, which is recognized by the Cas9 enzyme derived from *Streptococcus pyogenes*. The GC content was between 40% and 60%, the out-of-frame score, which is used to predict the likelihood of frame-shift mutations occurring upon indel formation in the target sequence, was set to at least 65, and the mismatch values were set to 1-0-0. Furthermore, GGCA was added to the 5′ end of the forward primer, and CAAA was added to the 5′ end of the reverse primer to ensure ligation with the *Bsa*I-digested pRGEB32 vector.

#### 4.2.3. Genome Editing in Rice Using CRISPR/Cas9 Vector Construction

A CRISPR/Cas9 vector was constructed to perform gene editing (Figure S1). After synthesizing the selected sgRNA, both forward and reverse sgRNAs were prepared at a concentration of 100 pmol. To synthesize the double-stranded sgRNA fragment, we prepared a total mixture of 50 µL by combining 5 µL of T4 ligase buffer, 5 µL each of forward and reverse sgRNA, and 35 µL of distilled water. The mixture was at 37 °C for 30 min and then heated at 94 °C for 5 min. Next, the temperature was gradually reduced to 30 °C in 1 °C intervals every 28 s, followed by incubation at 10 °C for 10 min. Subsequently, another mixture containing 1 µg of pRGEB32 vector, 1 µL of *Bsa*I, 5 µL of 10X CutSmart buffer (NEB), and distilled water was prepared to a final volume of 50 µL. The mixture was incubated at 37 °C for 15 min and then at 80 °C for 20 min to create sticky ends on the pRGEB32 vector. To ligate the *Bsa*I-digested pRGEB32 with the double-stranded gRNA, a mixture was prepared containing 2 µL of double-stranded gRNA, 2 µL of T4 ligase, 2 µL of T4 ligase buffer, and 50 ng of pRGEB32, with distilled water added to a final volume of 20 µL. The mixture was then incubated at 4 °C for 16 h. The final solution was transformed into Escherichia coli JM109 (TAKARA, Tokyo, Japan), plated onto solid Luria–Bertani (Bexton, Dickinson and Co., USA) medium containing kanamycin, and incubated in the dark at 37 °C for 16 h. To confirm the insertion of the sgRNA, a single colony was inoculated into a liquid LB medium containing kanamycin and incubated at 37 °C with shaking at 150 rpm for 16 h. Plasmid DNA was extracted using the QIAprep Spin Miniprep kit (QIAGEN, Cat. 69104, Hilden, Germany), and the sample was sent to Solgent (Solgent Co. Ltd., Daejeon, Republic of Korea) for sequencing. Based on the sequencing results, the sgRNA::pRGEB32 vector with the inserted sgRNA was selected and transformed into *Agrobacterium tumefaciens* EHA105 competent cells (TAKARA, Tokyo, Japan). *Agrobacterium tumefaciens* was cultured in a YEP medium containing rifampicin and kanamycin.

#### 4.2.4. Agrobacterium-Mediated Rice Transformation and Regeneration

Ilmi seeds were dehulled using a rice huller, and seeds with intact, white, and uncontaminated embryos were selected. The seeds were sterilized using 70% ethanol for 30 s under vigorous shaking, then treated with 1% NaOCl for 30 min using a reciprocal shaker. After washing the seeds three times with ddH_2_O, they were placed on sterile filter paper to dry. The dried seeds were then inoculated, ten per dish, on a callus induction medium (Table S3). Callus induction was performed in the dark at 25 °C for 21 days. After which, the husks and roots were removed, and hard, brightly colored calli, about 2 mm in diameter, were selected for a pre-culture period of 3 days. *Agrobacterium* containing the sgRNA::pRGEB32 plasmid were adjusted to an OD value of 0.8, and pre-cultured calli were immersed in the suspension and shaken for 1 min. The calli were then dried on sterile filter paper and cultured in a co-culture medium containing acetosyringone in the dark at 25 °C for 3 days. The calli inoculated with *Agrobacterium* were washed thrice for 30 min, each with ddH_2_O containing carbenicillin, followed by five washes with ddH_2_O. After drying with sterile filter paper, the calli were cultured on a regeneration medium at 26 °C under light conditions. We created subculture at 2-week intervals. Calli with green spots, which appeared during the transformation process, was transferred to a fresh regeneration medium, from which transgenic plants were obtained.

### 4.3. Molecular Analysis of Transgenic Plants

#### 4.3.1. DNA Extraction

Leaves of regenerated plants were frozen in liquid nitrogen and ground using a mortar and pestle. A total of 500 µL of DNA extraction buffer (2% CTAB, 0.1 M Tris, pH 8.0, 1.4 M NaCl, 1% polyvinylpyrrolidone) was added to 100 mg of the sample. After vortexing to mix, the sample was incubated at 65 °C for 20 min. After the reaction, PCI (phenol:chloroform:alcohol = 25:24:1) was added, and the mixture was gently inverted to mix. The mixture was left at room temperature for 20 min and then centrifuged at 13,000× *g* rpm and 4 °C for 10 min. After centrifugation, 400 µL of the supernatant was transferred to a new 1.5 mL tube, and 400 µL isopropanol and 40 µL of 3X sodium acetate were added. Again, the mixture was gently inverted to mix and centrifuged at 14,000× *g* rpm for 10 min. The supernatant was carefully discarded, and the pellet was washed using 70% ethanol. After centrifuging the washed pellet at 13,000× *g* rpm for 1 min, the supernatant was removed, and the pellet was air-dried on a clean bench for 10 min to remove any residual liquid. After air drying, 50 µL of ddH_2_O was added to dissolve the pellet. The DNA concentration was measured using a NanoDrop 2000 Spectrophotometer (ND-2000; Nanodrop, Waltham, MA, USA).

#### 4.3.2. RNA Extraction

After confirming the DNA insertion, the leaves of the surviving plants, excluding the four dead ones, were frozen in liquid nitrogen and ground using a mortar and pestle. A total of 1 mL of TRIzol reagent was added to 100 mg of the ground sample and mixed by vortexing, followed by incubation at room temperature for 10 min. The mixture was centrifuged at 12,000× *g* rpm and 4 °C for 10 min. The supernatant was transferred to a new tube, and 200 µL of chloroform was added. The mixture was inverted gently for 15 s to mix and then placed on ice for 3 min before being centrifuged again at 12,000× *g* rpm and 4 °C for 15 min. After centrifugation, the aqueous phase (upper part, about 400 µL) was transferred to a new tube. A total of 300 µL of isopropanol and 30 µL of 1.2 M NaCl were added, and the mixture was inverted gently to mix and left at room temperature for 10 min. The mixture was centrifuged at 12,000× *g* rpm and 4 °C for 10 min. After carefully discarding the supernatant, 1 mL of 75% ethanol was added to wash the pellet, and the mixture was centrifuged at room temperature and 10,000× *g* rpm for 5 min. After centrifugation, the supernatant was carefully discarded, and the mixture was centrifuged again in a small centrifuge for 30 s. The remaining supernatant was removed, and the pellet was air-dried on a clean bench for 10 min. The pellet was re-suspended in 50 µL DEPC water, and the RNA concentration was measured using a NanoDrop 2000 Spectrophotometer. Finally, RNA samples were stored at −20 °C until required.

#### 4.3.3. cDNA Synthesis

cDNA synthesis was performed using the UltraScript 2.0 cDNA Synthesis kit (PCRBIOSYSTEMS, Wayne, PA, USA), following the manufacturer’s instructions. A master mix was prepared by combining 4 µL of 5× cDNA Synthesis Mix, 1 µL of 20× RTase, 1 µL of 100 ng/µL RNA, and ddH_2_O to a final volume of 20 µL. The master mix was incubated at 50 °C for 30 min and then at 95 °C for 10 min to synthesize cDNA.

#### 4.3.4. Genotype Analysis of Regenerated Plants

To confirm the genes had been inserted in the regenerated plants, PCR was performed to amplify *OsActin*, the target gene *OsDGTq1*, and the *Cas9* and *hygromycin resistance* (*HPT II*) genes in the T-DNA region of pRGEB32 (Table S4). Electrophoresis PCR was performed using Primer Taq DNA polymerase (GENETBIO, Daejeon, Republic of Korea), and the bands were confirmed. A master mix was prepared by mixing 100 ng of genomic DNA, 2 µL of forward and reverse primers (10 pmol each), 2 µL of 10× reaction buffer, 1 µL of dNTP, and 0.1 µL of Taq polymerase. The final volume was adjusted to 20 µL by adding distilled water. The PCR reaction conditions consisted of an initial denaturation step at 95 °C for 5 min, followed by 35 cycles for denaturation at 94 °C for 30 s, annealing at each primer’s recommended temperature for 30 s, and extension at 72 °C for 1 min, the final extension was performed at 72 °C for 5 min. After the PCR, the products were separated on a 0.8% agarose gel. The band representing the target gene *OsDGTq1* was excised and purified using the QIAquick Gel Extraction kit (QIAGEN, Cat. 27206, Hilden, Germany). Sequencing chromatogram analysis was performed and compared with the Ilmi sequence.

#### 4.3.5. Analysis of Gene Expression in Regenerated Plants

Using the synthesized cDNA, the expressions of *OsActin*, *Cas9*, *HPT II*, and the target gene *OsDGTq1* were confirmed in the regenerated plants. The same primers used for genotype confirmation were employed, and the reaction was performed using Primer Taq DNA polymerase. The reaction consisted of an initial denaturation step at 95 °C for 5 min, followed by 35 cycles comprising denaturation at 94 °C for 30 s, annealing at each primer’s recommended temperature for 30 s, and extension at 72 °C for 1 min. A final extension was performed at 72 °C for 5 min. After the PCR, the products were separated on a 0.8% agarose gel.

#### 4.3.6. Investigation of Agricultural Traits in Transgenic Lines

The transgenic plants were cultivated in the greenhouse at Kyungpook National University. The line names were assigned prior to investigating agronomic traits. The agronomic traits of the *OsDGTq1*-G_0_ lines and Ilmi, including culm length (cm), panicle length (cm), the number of tillers and the number of spikes were investigated. The plant height was measured from the ground to the tip of the panicle. The culm length was measured from the ground to the panicle nodreace, and the panicle length was measured from the node to the tip of the panicle. The number of primary tillers was investigated. The seed setting rate was evaluated using the Single-seed-decent method.

#### 4.3.7. Analysis of Gene Expression Levels in the Genome-Edited Rice

Quantitative real-time PCR (qRT-PCR) was performed to investigate the expression levels of *OsDGTq1*, *Cas9* and *HPT II* in Ilmi, *OsDGTq1* G1, *OsDGTq1* G2, and *OsDGTq1* G3. The qPCR mixture was prepared using BioFact™ 2X Real Time PCR Master Mix (Cat No. DQ383-40H, BIOFACT Co., Ltd., Daejeon, Republic of Korea). Each reaction mixture contained 10 µL of 2X Real-Time PCR Master Mix, 1 µL of forward primer (10 pmol/µL), 1 µL of reverse primer (10 pmol/µL), 1 µL of cDNA, and ddH2O to a final volume of 20 µL. The qRT-PCR was conducted using the Eco Real-Time PCR System under the following conditions: initial denaturation at 95 °C for 2 min, followed by 40 cycles of denaturation at 95 °C for 20 s, annealing and extension at 58 °C for 40 s. The final melting curve analysis was performed at 95 °C for 15 s, 55 °C for 15 s, and 95 °C for 15 s. *OsActin* was used as the housekeeping gene. The relative expression levels were calculated using the comparative 2^−ΔΔCt^ method, and each reaction was performed in triplicate.

#### 4.3.8. Inoculation with Damping-Off Pathogens in Leaves of Genome-Edited Plants

To compare and analyze lesion lengths, the damping-off pathogens were inoculated onto the third leaf of genome-edited rice. For the parental plant to analyze lesion lengths, Ilmi, the background variety for the development of the genome-edited rice. After isolating *P. graminicola* and *R. solani* on solid media, the mycelia were inoculated into potato dextrose broth and cultured under dark conditions at 25 °C for 3 days at 150 rpm. After dipping the scissors in the culture solution, the third leaf was cut to inoculate the strain.

#### 4.3.9. Statistical Analysis

For statistical analysis, three independent trials were conducted. In each trial, phenotypic data were collected from ten randomly selected plants per line, including genome-edited plants, and subjected to statistical evaluation. Statistical evaluations were performed using SPSS statistical software (IBM SPSS Statistics, version 22, version 26, Redmond, WC, USA). To compare the mean values among lines, a *t*-test and one-way analysis of variance (ANOVA) were performed, and statistical significance was evaluated at the *p* < 0.05 level. Multiple comparisons were conducted using Duncan’s Multiple Range Test.

## Data Availability

The original contributions presented in this study are included in the article/Supplementary Materials. Further inquiries can be directed to the corresponding author.

## Supplementary Materials

The following supporting information can be downloaded at: https://www.mdpi.com/article/10.3390/ijms26199761/s1.
