# Supplementary material for: Development of Resistance to Damping-Off in Rice, Oryza sativa L., Using CRISPR/Cas9
Source: Int J Mol Sci. 2025 Oct 7;26(19):9761. doi: 10.3390/ijms26199761 (PMC12525438; doi:10.3390/ijms26199761)
Supplement: Supplementary file 1 [file ijms-26-09761-s001.zip › ijms-3863211-supplementary.pdf]

Supplementary Materials for:

Development of Resistance to Damping-Off in Rice, *Oryza sativa* L., Using CRISPR/Cas9

Table S1. Investigation of G_0 *OsDGTq1* transgenic plants

Accession number	Plant height (cm)	Culm length (cm)	Panicle length (cm)	No. of tillers	No. of spikes
Ilmi	59	46	13	7	7
OsDGTq1-G1	52	37	15	10	10
OsDGTq1-G2	53	40	13	5	4
OsDGTq1-G3	51	35	16	7	5
OsDGTq1-G4	50	35	15	5	5
OsDGTq1-G5	52	36	16	4	4
OsDGTq1-G6	51	38	13	6	6
OsDGTq1-G7	49	37	12	8	7
OsDGTq1-G8	53	48	15	5	5
OsDGTq1-G9	45	32	13	11	5
OsDGTq1-G10	55	41	14	5	5
OsDGTq1-G11	49	32	17	5	5
OsDGTq1-G12	51	37	14	11	10
OsDGTq1-G13	49	33	16	8	8
OsDGTq1-G15	44	30	14	7	7
OsDGTq1-G16	48	31	17	6	6
OsDGTq1-G17	45	32	13	6	5
OsDGTq1-G18	57	40	17	5	5
OsDGTq1-G19	43	31	12	5	5
OsDGTq1-G20	55	25	30	5	5
OsDGTq1-G21	43	30	13	6	3
OsDGTq1-G22	57	39	18	8	8
OsDGTq1-G23	49	35	14	10	7
OsDGTq1-G24	47	31	16	5	4
OsDGTq1-G25	52	37	18	5	4
OsDGTq1-G26	52	35	13	7	7
OsDGTq1-G27	51	35	16	6	6
OsDGTq1-G28	49	32	17	8	8
OsDGTq1-G29	65	45	20	9	6
OsDGTq1-G30	59	40	19	5	5
OsDGTq1-G31	59	35	24	7	7
OsDGTq1-G32	60	40	20	7	7
OsDGTq1-G33	58	42	16	5	4
OsDGTq1-G34	64	42	22	7	7

Table S2. Investigation of G_0 fertile grains of *OsDGTq1* transgenic rice

Plant No.	No. of total grains	No. of fertile grains	No. of unfertilized grains	Percent of fertile grains (%)
OsDGTq1-G1	58.2±31.5[z]	0.2± 0.6	58.0±31.5	0.4± 1.4
OsDGTq1-G2	63.0±38.0	0.3± 0.5	62.8±37.9	0.3± 0.5
OsDGTq1-G3	60.2±19.0	0.0± 0.0	60.2±19.0	0.0± 0.0
OsDGTq1-G4	56.8±20.2	45.5±18.7	10.3± 3.3	80.8± 5.7
OsDGTq1-G5	57.8±17.9	50.0±17.7	7.8± 1.5	85.7± 4.7
OsDGTq1-G6	62.8±20.9	53.0±18.3	9.8± 4.6	84.4± 7.0
OsDGTq1-G7	47.8±22.5	37.0± 9.6	10.8±18.3	84.2±19.2
OsDGTq1-G8	44.8±14.5	35.5± 7.7	9.3± 8.0	81.4±10.2
OsDGTq1-G9	50.8±10.3	7.4± 5.9	43.4± 8.8	14.2±11.6
OsDGTq1-G10	72.2±24.9	0.2± 0.4	72.0±25.3	0.7± 1.5
OsDGTq1-G11	41.6±20.2	17.8±17.0	23.8±11.2	36.0±34.9
OsDGTq1-G12	50.5±18.7	16.9±12.3	33.6±12.0	29.5±20.5
OsDGTq1-G13	45.6±19.8	18.2±16.3	27.4± 7.3	32.8±22.9
OsDGTq1-G15	49.8±20.8	0.4± 1.1	49.4±21.0	0.9± 2.5
OsDGTq1-G16	57.9±13.7	20.6± 9.7	37.3±17.0	37.7±21.0
OsDGTq1-G17	43.8±16.7	27.8±18.3	16.0± 8.0	59.4±21.7
OsDGTq1-G18	94.6±15.6	12.0±12.2	82.6±25.5	14.2±16.1
OsDGTq1-G19	44.6± 8.2	11.6±11.5	33.0± 4.5	23.0±22.0
OsDGTq1-G20	67.2±23.4	10.2± 3.5	57.0±22.4	16.7± 6.8
OsDGTq1-G21	54.8±25.3	8.0±10.2	46.8±32.7	21.0±25.8
OsDGTq1-G22	64.8±19.5	9.2±13.8	55.6±20.7	13.0±20.7
OsDGTq1-G23	59.6±18.2	1.4± 3.2	58.3±19.1	2.6± 5.9
OsDGTq1-G24	52.0±34.0	4.3± 3.0	37.7±35.4	16.9±14.3
OsDGTq1-G25	126.0±14.0	0.0± 0.0	126.0±14.0	0.0± 0.0
OsDGTq1-G26	81.8±28.1	26.5±17.0	55.3±30.3	33.5±18.2
OsDGTq1-G27	65.1±28.1	26.3± 8.8	38.9±22.1	42.4± 8.5
OsDGTq1-G28	70.2±16.9	24.5±10.3	45.7±19.5	36.4±18.1
OsDGTq1-G29	66.3±14.7	5.3± 6.3	61.0±13.0	7.3± 8.9
OsDGTq1-G30	57.5± 9.6	8.2± 2.1	49.3±11.2	15.0± 6.5
OsDGTq1-G31	57.6±28.8	3.2± 7.2	54.4±34.0	13.9±31.1
OsDGTq1-G32	75.7±11.6	20.0±14.1	55.7±20.0	27.2±21.6
OsDGTq1-G33	79.7±32.7	0.7± 1.2	79.0±33.9	1.6± 2.7
OsDGTq1-G34	60.8±27.3	5.0± 5.7	55.8±25.1	8.3± 6.8

zmean±standard deviation

Table S3. Composition of each medium for plant regeneration of genome-editing in rice

Medium	Composition
Callus induction	4.4 g/L MS powder (Murashige and Skoog, 1962), 30 g/L sucrose, 2.878 g/L proline, 300 mg/L casein hydrolysate, 3 mg/L 2,4-D, 4 g/L gelrite, 100 mg/L myo-inositol, pH 5.8
Co-cultivation	4.4 g/L MS powder (Murashige and Skoog, 1962), 30 g/L sucrose, 2.878 g/L proline, 300 mg/L casein hydrolysate, 3 mg/L 2,4-D, 4 g/L gelrite, 100 mg/L myo-inositol, 100 mg/L acetosyringone, pH 5.2
Regeneration	4.4 g/L MS powder (Murashige and Skoog, 1962), 30 g/L sucrose, 30 g/L D-sorbitol, 300 mg/L casein hydrolysate, 2 mg/L Kinetin, 1 mg/L NAA, 4 g/L gelrite, 100 mg/L myo-inositol, 500 mg/L cefotaxime, 50 mg/L hygromycin, pH 5.8
LB	25 g/L LB powder (Luria-Bertani), 50 mg/L kanamycin, 4 g/L gelrite
YEP	10 g/L yeast, 10 g/L peptone, 5 g/L NaCl, 50 mg/mL rifampicin, 50 mg/L hygromycin, 6 g/L agar
PDA	24 g/L Potato Dextrose broth, 15 g/L agar, pH 5.2

Table S4. Primer set for analysis of expression level and sequencing in genome-editing rice

Primer name	Forward/Reverse	Sequence (5’ to 3’)
OsActin	Forward	TGAATCTGGTCCAGGCATCG
	Reverse	TGGGACGCATGCAAACAATC
OsDGTq1	Forward	CCATGTGCTGTTTGTGACGG
	Reverse	TTCGGAATTCGATGCCACCA
Cas9	Forward	ACAAGCTGATCCGGGAAGTG
	Reverse	ACCAGCACAGAATAGGCCAC
HPT II	Forward	GATGCCTCCGCTCGAAGTAG
	Reverse	TTGGGGAGTTTAGCGAGAGC
*OsActin*_qPCR	Forward	ACCACAGGTATTGTGTTGGAC
	Reverse	AGAGCATATCCTTCATAGATGG
*OsDGTq1*_qPCR	Forward	GAATCCGAAAGCTGAGCGTG
	Reverse	TCGTAATCTGTGAGCTGCCA
*Cas9*_qPCR	Forward	GAGAACCAGACCACCCAGAA
	Reverse	GCTCTTTGATGCCCTCTTCG
*HPT II*_qPCR	Forward	CCCCAATGTCAAGCACTTCC
	Reverse	GCGCCGATGGTTTCTACAAA
OsDGTq1-sgRNA1	Forward	GGCATCCGGCTTGGCGAATGCTTT
	Reverse	AAACAAAGCATTCGCCAAGCCGGA
OsDGTq1-sgRNA2	Forward	GGCACAGCAGCTTCCAAGCCCTTC
	Reverse	AAACGAAGGGCTTGGAAGCTGCTG
OsDGTq1-sgRNA3	Forward	GGCATCCTACAATGGTGGTGACCA
	Reverse	AAACTGGTCACCACCATTGTAGGA

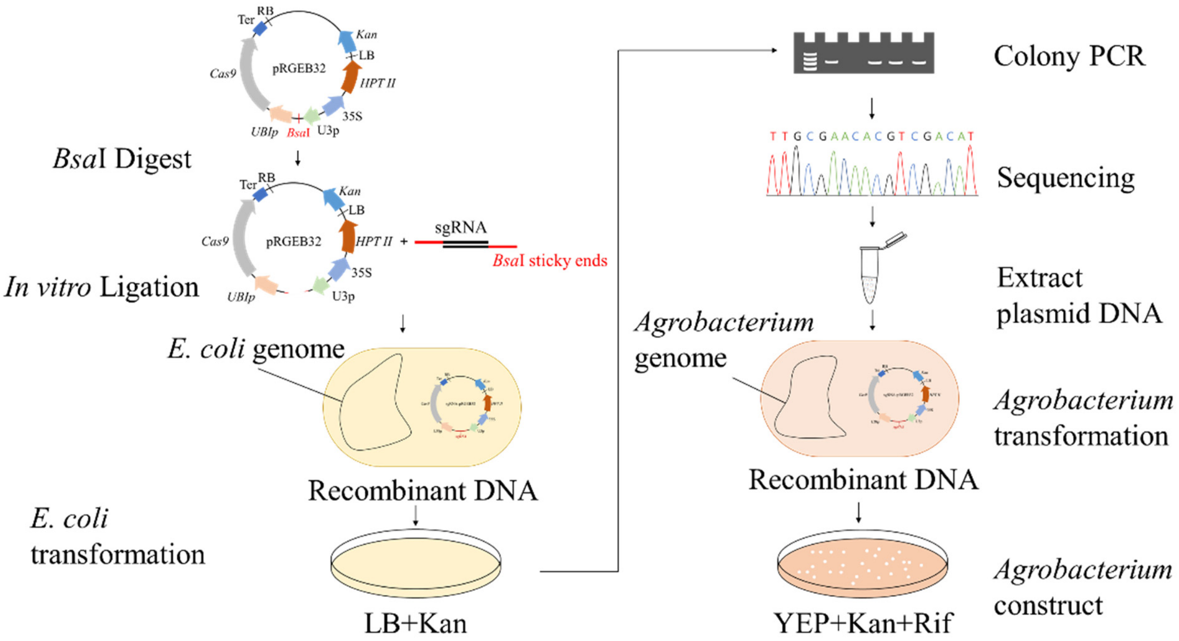

Figure S1. **Construction of a CRISPR/Cas9 vector for genome editing in rice.** A single guide RNA (sgRNA) was inserted into the *Bsa*I restriction site of pRGEB32. *Bsa*I treatment generated sticky ends, allowing for the specific ligation of gRNAs designed to be complementary to these ends. The recombinant plasmid was transformed into *E. coli* and cultured in a LB medium with kanamycin. Plasmid DNA containing correctly inserted sgRNA was selected by colony PCR and sequencing, then transformed into *Agrobacterium*, which was cultured in a YEP medium supplemented with rifampicin and kanamycin.
